# Results of glycated hemoglobin during treatment with insulin analogues dispensed in the public health system of Federal District in Brazil

**DOI:** 10.1186/s13098-015-0061-0

**Published:** 2015-08-18

**Authors:** Eliziane Brandão Leite, Hermelinda Cordeiro Pedrosa, Luiz Augusto Casulari

**Affiliations:** Diabetes Central Coordination, State Health Secretariat of Federal District, SQS 304 Bloco F Apto 403, Brasília, DF 70337-060 Brazil; Research Centre, Foundation of Teaching and Research on Health Sciences, Brasília, DF Brazil; Unit of Endocrinology, Taguatinga Regional Hospital-HRT, Brasília, DF Brazil; Endocrinology Service, University Hospital (HUB) and University of Brasília (UnB), Brasília, DF Brazil

**Keywords:** Diabetes, Insulin Analogues, Glargine, Glycated Hemoglobin, Treatment

## Abstract

**Background:**

Diabetes treatment requires specialized multi-professional teams, supplies for blood glucose monitoring and training for self-injections of human insulin or insulin analogues. The State Health Secretariat of the Federal District (SHS-FD) has dispensed insulin analogues by means of clinical validated protocols since 2004. However, data on outcomes of follow-up are still unknown.

**Objective:**

To evaluate the results of glycated hemoglobin (HbA1c) among diabetic patients treated with insulin analogues.

**Methods:**

It is a retrospective cohort study involving data of type 1(DM1) and type 2 diabetes (DM2) patients 18 years old and above who were registered to participate at the insulin analogues dispense program of the SHS-FD. Evaluation of criteria of insulin treatment continuity was based on HbA1c values achieved in the follow-up period: *in the target*, <7 %, patients between 18 and 65 years old; <8 % for those above 65 years old; *out of target*, when values were superior these cut off points for both age groups; and *minimum 0.5* *% reduction* of two HbA1c values during follow-up.

**Results:**

Two hundred and fifteen formularies were analyzed: Type 2 patients (63.7 %) and female sex were the most prevalent (63.7 %), (p < 0.05). Mean age and SD were 41.5 ± 23.5 years among DM1 and 60.5 ± 28.5 in those with DM2. HbA1c *in the target* was found in 26 %, 48 % were *out of target* and 26 % achieved *0.5* *% minimum reduction* in HbA1c value (p < 0.05). The main clinical characteristics associated with HbA1c found to be *in the target* were older age (>65 years), more than three medical appointments in the follow-up and lower mean HbA1c in the patient selection for inclusion criteria in the dispense program (p < 0.05).

**Conclusion:**

The low number of patients using insulin analogues *in the target* group, considered to be in good control, implies the need to reevaluate both level of patients self-care knowledge and glucose monitoring prior their inclusion in the insulin analogue dispense program. Reinforcement and training of health professional teams in enrollment procedures should be on mandatory basis to avoid protocol failure or deviations.

**Electronic supplementary material:**

The online version of this article (doi:10.1186/s13098-015-0061-0) contains supplementary material, which is available to authorized users.

## Background

Insulin analogues have been dispensed to both type 1 and type 2 diabetes patients for the last 10 years by the State Health Secretariat of the Federal District (SHS-FD) in Brazil [1, 2]. The cost is high and covered by the Federal District Government budget. Insulin analogue dispense is based upon a validated clinical protocol, which has been regularly updated [2–6] and involves clinical features and glycated hemoglobin (HbA1c) targets to be analyzed every 6 months by the medical team before being renewed. Currently, two long acting insulin analogues have been dispensed, glargine and detemir, and one of short acting analogues (lispro, aspart or glulisin).

Type 2 (DM2) treatment approach is by primary care teams of SHS-FD network and includes clinical and education besides pharmaceutical supervision to deliver supplies and medications to all patients. Secondary and tertiary specialized health professional teams are responsible to manage the care and therapy interventions for all DM1 patients and those on the continuous insulin infusion system (CIIS), DM2 patients who are mainly on insulin treatment, pregnant diabetic women, diagnosis procedures and follow-up of diabetic long term complications.

SHS-FD has long been evolving in the standard of care and assistance to diabetic patients since the Program of Education and Control of Diabetes (PECD) was set up in 1988 [7]. In 1994, a local law named *Lei Distrital 640* empowered the PECD coordinators to implement important policies on primary care grounds including educational seminars for the health care providers to be able to educate patients at health centers. Moreover, the law claimed mandatory human insulin dispense, syringes, glucose strips and meters, all has been in the pharmaceutical portfolio since 1995. Long acting analogue glargine use had been previously used at a hospital unit of SHS-FD that participated as center of a national study [8]. Its positive result contributed to be available in 2005, right after short acting lispro (2004) and later, detemir (2007) [9].

These insulin analogues have not been approved to be dispensed by the Brazilian Ministry of Health which only registered intermediate acting human insulin NPH (*neutral protamine Hagerdon*) for basal therapy and short acting (rapid, regular) in RENAME (Relação Nacional de Medicamentos, National Medicines List), its official portfolio [10].

It is well recognized the main positive and proved effects of insulin analogues on hypoglycemia reduction, glucose peaks and improvement in quality of life [1, 11–13] of DM1 patients. Recent meta-analysis have also reinforced long acting efficacy, safety and positive cost effects with long acting insulins over NPH [14, 15].

Importantly to mention that, despite being dispensed in more than ten states in the country, there are no direct reports focusing the current situation of diabetic patients treated in the Brazilian Unified Health System (hereby SUS) with long and short acting insulin analogues. Therefore, this study aims to evaluate the glycemic control by means of HbA1c results achieved by the registered patients treated with insulin analogues in the SUS of Federal District (SUS-FD).

## Research design and methods

It is a retrospective cohort study involving DM1 and DM2 patients registered at Hospital Regional do Gama (HRG), a health district of SHS-FD, to use insulin analogues, in the period of 2005–2012. It was approved by the Ethical Committee of the Foundation of Teaching and Research in Health Sciences (FEPECS, Foundation for Teaching and Research in the Health Sciences) of SHS-FD.

All registered inclusion and continuity forms for using insulin analogues and renewing every 6 months, since enrollment start in 2005, was collected from January to December 2013.

Sample involved active users of insulin analogues who had at least two HbA1c (High Perfomance Líquid Cromatography method) results after initiating treatment, aging 18 years old or above, from either public or private health system. Exclusion criteria were pregnancy, hemoglobinopathy, insulin analogue detemir use (small dispense number of this other available long acting analogue) and for multivariate analysis, twenty-nine patients without using short acting insulin analogue were excluded from study.

Three groups of patient were set based on HbA1c: *in the target*, <7 % for diabetic patients between 18 and 65 years old; <8 %, for those above 65 years old; *out of target*, values superior to cut off points for both age groups; *minimum 0.5* *% reduction,* difference between of two HbA1c values achieved during follow-up with 6 months interval. If any medical reason was found to explain a given patient not to reach the goal, he or she could be maintained in the analogue therapy until next scheduled clinical visit.

Statistical tests verified patient profile to HbA1c values as results of insulin analogues use. Mean, maximum, minimum and standard deviation (SD) were calculated. Chi square test (X^2^) to access categorical variables and Student *T* test to compare means, with 5 % statistical significance. Multivariated analysis aimed to identify how selected variables related to HbA1c values during last evaluation; linear model without distinction of DM type. In order to reach better estimates, it was applied a robust model such as White’s *heteroscedasticidade.* Software Stata version 2.0 model was used for all tests. The estimated model is shown below:

$$ {\text{HbA1c}} = \alpha + \beta_{1} {\text{sex}} + \beta_{2} {\text{age}} + \beta_{3} {\text{combination}} + \beta_{4} {\text{insulin}} + \beta_{5} {\text{treatment duration}} + \beta_{6} {\text{exam amount}} + \varepsilon $$

where HbA1c is the last value, α is the constant, Sex is the Duma, signalizes patient sex: 1—for female; 0—for male, *Age* is the patient age obtained at last evaluation, *Combination* is the dummy, signalizes if patient is treated with combination of types of insulin analogues (short acting and glargine) or if he or she uses only one type. 1, combination, 0 if an exclusive type is used, *Insulin* is the total dose of insulin used by patient. If combination of short acting and glargine was used, total dose was sum of quantity of units of each insulin type, *Duration of treatment* is the time that patient has been under treatment, based on the admission year in the programe, *Quantity of exams* is the quantity of appointments when HbA1c was verified, ε is the Model residual.

## Results

Two hundred and fifteen diabetic patients were using insulin analogues at the HRG. As shown in Additional file 1: Table S1, DM1 was diagnosed in 78 (36.3 %) and DM2 in 137 (63.7 %). Majority was female and they were diagnosed as DM2 compare to DM1 (p = 0.02). Mean age and SD were 41.5 ± 23.5 years for DM1 and 60.5 ± 28.5 years old for DM2 patients. Age stratification (18 to 34 and 35 to 64 years old) showed similarities in DM1 group, and none was above 65 years old. DM2 patient distribution in the age groups from 35 to 64 years old and above 65 years old were similar, and there was only one patient between 18 to 34 years old. Disease duration for both DM types over 10 years was the most prevalent.

It is also shown in Additional file 1: Table S1, majority of sample took 31–60 UI of glargine insulin. A few DM1 users reported high glargine doses, unit amount ranged from 8 to 120 UI/day. Short acting insulin below 30 UI was the most common dose (88.1 % in DM1 group and 69.7 % in DM2 group), and dose ranged from 3 to 102 UI representing sum of insulin injections during the day. Dose register lacked in 29 patients. Proportion of basal–bolus regime (50–60 % of basal insulin and short acting 50–40 %), was verified in 64.4 % DM1 and in 50 % DM2 patients. Glargin insulin and short acting combination was found in 84 % of patients, while only glargin and short acting single use was observed in 14 and 2 %, respectively.

Additional file 2: Table S2 shows HbA1c results in relation to age, sex, insulin use and continuity criteria in both DM1 and DM2 patients. Mean HbA1c study sample was 8.9 % (9.1 % for DM1 and 8.7 % for DM2). A significant difference of HbA1c was observed *in the target* group compare to the following situations: higher in those with a *0.5* *% HbA1c minimum reduction* (p = 0.05); values were lower in DM1 women compare to those with DM2 (p = 0.01). There were no statistical difference in other analysis.

In Additional file 3: Table S3 is shown the frequency of DM1 and DM2 according to sex and based in the continuity criteria of insulin analogue use. Minority of patients was *in the target* (26 %) or *0.5* *% minimum reductionin* HbA1c (26 %) (p = 0.001), most of them was *out of target* (48 %). There are significant percentage difference *in the target* group and those patients in *out of target* group (p = 0.001), *out of target* group compare to *0.5* *% minimum reduction* of HbA1c values (p = 0.001) as well. On the other hand, patients *out of target* group versus those of *0.5* *% minimum reduction* group demonstrated no significant difference in the HbA1c results (p = 0.89). DM1 shows a significant lower initial mean HbA1c *in the target* group compare to those *out of target* (p = 0.03) and in the *0.5* *% minimum reduction* individuals (p = 0.003). There is a significant reduction of final HbA1c value compare to the initial HbA1c value (p = 0.01), no specific difference related to sex was verified only in those *in the target* group. As for DM2, initial mean HbA1c *in the target* group was significantly lower than in *out of target* (p = 0.009) group, and among those presenting *0.5* *% minimum reduction* of HbA1c (p = 0.001).

Additional file 4: Table S4 shows HbA1c multi-variation analysis of 186 patients concerning sex, age, insulin combination treatment, total insulin dose, treatment duration and quantity of available exams. There is a positive relation among women who showed 0.63 % increment in the HbA1c values (p = 0.03). Age was a contributing factor favoring HbA1c lower values: 0.02 % significant reduction for each additional year (p = 0.01). Number of medical appointments also resulted in 0.26 % reduction of HbA1c (p = 0.01), as per appointment.

## Discussion

This is a pioneer study, to our knowledge, which evaluates results HbA1c of DM1 and DM2 individuals under treatment with insulin analogues dispensed in the SUS of Brazil. This research was performed at a hospital with a diabetes specialist team, providing secondary care in Federal District south region which comprising nearby cities from the Brazilian states of Minas Gerais and Goiás.

Since this is a retrospective study, it was not possible to access the socio-demographic data of patients, if from public or private sector, because these items were not included in the forms. Evaluation of diabetes education approach, that is part of the routine health care provision, was not an objective of the study.

Majority were women (63.7 %), particularly in the DM2 group (69.3 %). It is important to emphasize that other Brazilian reports also show women to adhere to most of types of treatment [16], although it is not a finding in other studies [7, 17], which shows similar frequency of men and women as participants. There were an increased number of older DM2 individuals and it is in accordance to other population studies involving both types of DM [18]. Diagnosis duration was lower in DM1 group compare to DM2 group.

Insulin therapy only is one of main criteria to dispense insulin analogues in the SHS-FD protocol [2] and that was present in 84 % while 14 % used only insulin glargine. That is a disagreement to enroll patients in the protocol. However, short acting insulin only is justified for continuous insulin infusion system (CIIS) or with NPH insulin, which comprised 2 % of sample.

Higher insulin doses of glargine and short acting analogues (30 UI or more), as expected, were shown in the DM2 group. In a review study, mean glargine dose was 30 UI [12], while in randomized control trials (RCT) a lower mean of 29.6 UI was verified [19]. A balance of glargine and short acting insulin doses was present in only 50 % of patients. That might have influenced HbA1c results.

Mean HbA1c was 8.9 %, lower than another Brazilian study whose mean was 9.1 and 40 % presented HbA1c above 9 % [20]. In the DM2 group, HbA1c mean was 8.7 %, similar to HbA1c of a recent meta-analysis (8.6 %) [21]. Moreover, high HbA1c values during glargine treatment have not been observed in other studies: 7.9 % [19], 7.1 % [22] values have been reported for DM2 while another study verified 8.0 % for DM1 [23].

There are controversies about the superiority of basal analogues to NPH therapy for DM2 [23] and while some reports have shown larger HbA1c decrease under glargine use [14, 15, 22] others have found no superiority of glycemic control with analogues [21, 24].

In the present study *0.5* *% minimum reduction* of HbA1c was observed among 26 % of patients, which is the minimum cut off point for renewing and dispensing insulin analogue in the SHS-FD protocol [2, 25, 26]. Other authors have considered a 1.0 % minimum reduction of HbA1c for a successful target of glargine use [27].

Protocol positive therapeutic response based on individual’s age [28]. Thus, <7 % for individuals between 18 and 65 years old and <8 % over 65 years old were achieved by 26 % of the whole sample and it was more frequent in DM2 (31.4 %) than in DM1 (16.7 %) group. These results are lower than other authors who showed HbA1c <7 % in 50.3 % DM2 [22] and 57.7 % in DM2 patients plus cardiovascular risks [27].

In a cross-sectional multicenter Brazilian study, only 13.2 % [20] and 10 % [16] of DM1 patients were in the target of HbA1c <7 % by means of different types of insulin therapy. In this study, frequency of those DM1 under insulin analogue treatment in the target was higher (16.7 %) which suggests a real improvement after treatment with glargine insulin. Another report showed that glargine use was similar to NPH, mixed insulin or lispro to achieve HbA1c less than 7 % in DM2 patients not well controlled with oral agents [24].

Majority of study patients did not achieve HbA1c target in the expected time before renewing dispense. Protocol allows maintenance of analogue continuity if special medical situations are related. It was verified *in the target* group initial lower HbA1c than both *out of target* or *0.5* *% minimum reduction* groups. Previous reports show better control among diabetic patients who presents lower initial HbA1c [24].

Statistical model F test applied for multivariate analysis shows that all variables taken together explain variations verified in the HbA1c levels. Model can only explain 14 % variation that influenced HbA1c and was not included in the multivariate analysis. Patients well enrolled in program proved benefit of continuity for achieving HbA1c targets and potentially hypoglycemia reduction and better quality of life. Indeed, other studies have shown the necessity of evaluating glycemic variability [29, 30], individualizing targets [31], and warning difficulty of treatment and adherence as HbA1c only does not explain the complex factors of a success control [32].

Costs of treatment demands attention by health policymakers and managers. Lower glargine cost than NPH has been described in DM2 [33], but recent systematic review whose focus is mainly to point out costs [34], has suggested that analogue insulins should not be dispensed in Minas Gerais, a state in the southeast of Brazil, or if so, it should request a negotiation with pharmaceutical industry to reduce its price. Authors argue its price and difference to NPH cost have increased 291 and 536 % in recent years, respectively. They quote majority of glargine studies to be of “poor methodological quality or had high risk of bias”, and “there was no significant difference between glargine insulin and NPH insulins” [34]. That review article did not consider other aspects of glargine treatment. According to another author [35], “it seems inappropriate and flawed from the start as studies on glargine insulin began in the late 1990s and not in 1970 besides data have been published since 2009”. Claims that five of eight papers selected for analysis were of poor quality and had risk of bias; one article compares two forms of insulin analogue delivery and not NPH; another study had only 4 month duration; and hypoglycemia reduction, a gold-standard outcomes of insulin analogues versus NPH, was not analyzed were also pointed out.

This study shows some limitation as it is a retrospective research and has not evaluated hypoglycemia. The HRG-SHS/FD health district selected for this data analysis does not represent the entire socio-economical parameters of Federal District, neither the clinical practice reality of other health districts. These factors might have influenced results and enrollment was inappropriate due the higher number of DM2 patients compare to DM1. That probably has biased the sample although protocol includes DM2 in special clinical situations and conditions only. Higher frequency of DM2 women also possibly influenced results related to gender.

Thus, clearly it is necessary to evaluate how health professionals have been enrolling participants and mandatory recall for protocol training as well. That potentially shall turn into a proper dispense and better surveillance of the insulin analogue program and provide means to a more adequate clinical follow-up. On the other hand, re-evaluation of self-care and SBGM should be carefully tailored among patients because dispense of insulins (of any type), glucose meters and strips seem not to be the main strategies unless education is provided, as these issues have already been showed in other Brazilian studies on both DM1 and DM2 [16, 20].

This was a pioneer real-life study with insulin glargine users and short acting analogues in the Brazilian SUS. It is valid to mention that patients were included at different times from the year 2005, when insulin glargine launched in the Brazilian market and a multicenter study had finished in the country with positive results [8]. Later on, the preliminary Federal District experience reported the very first version of the SHS-FD protocol [9]. Recently, Brazilian Ministry of Health also had to evaluate the inclusion of analogue insulins in its RENAME list [10], after a public audience. Its reports based on RCT, meta-analysis and mainly on glucose control and cost: decision was contrary to dispense new insulins in the SUS, even though recent robust reports clearly favor its use for DM1 [14, 15].

In conclusion, insulin analogue use at HRG-SHS/FD showed a little over than a quarter of patients achieved protocol glycemic targets of HbA1c <7.0 % and the same proportion had 0.5 % reduction. That implies the need to reevaluate both patients level of self-care knowledge and SBGM to be included in the dispense program, to detect early individual difficulties and limitations in order to provide education, proper and regular surveillance by health professionals. Potential adjustments and improvement of participant enrollment in the SHS-FD protocol, based on the present data and study limitations especially on larger DM2 inclusion, shall favor better glycemic control and quality of life in the near future and positive impact in the cost-benefit ratio of this pioneer national insulin analogue dispense program of the SUS-FD.

## Additional files

Additional file 1:
**Table S1.** Distribution of 215 active diabetic users of insulin analogues related to sex, age, duration of diabetes diagnosis and insulin dose. Additional file 2:
**Table S2.** Mean, standard deviation (SD) of glycated hemogoblin (HbA1c) related to age, sex, type of insulin and targets to continuity of treatment for type 1 and type 2 diabetes. Additional file 3:
**Table S3.** Frequency of type 1 and type 2 diabetes among female and male according to criteria of continuity of insulin analogue treatment, based in HbA1c range of values. Additional file 4:
**Table S4**. Multivariate analysis without distinction of diabetes type, HbA1c with sex, age, insulin combination, total dose of insulin, duration of treatment, and quantity of exams in 186 diabetic patients.

## Abbreviations

CIIS: continuous insulin infusion
DM1: type 1 diabetes
DM2: type 2 diabetes
HgbA1c: glycated hemoglobin
HRG: Hospital Regional do Gama
HRG-SHS-FD: Hospital Regional do Gama- State Health Secretarial of Federal District
Insulin NPH: neutral protamine Hagerdon
PELD: program of education and control of diabetes
RENAME: National list of medicines
SD: standard deviation
SHS-FD: State Health Secretarial of Federal District
SUS: Brazilian unified health system
SUS-FD: Brazilian unified health system of Federal District
