# Supplementary material for: Results of glycated hemoglobin during treatment with insulin analogues dispensed in the public health system of Federal District in Brazil
Source: Diabetol Metab Syndr. 2015 Aug 18;7:66. doi: 10.1186/s13098-015-0061-0 (PMC4539715; doi:10.1186/s13098-015-0061-0)
Supplement: Additional file 1: — Table S1. Distribution of 215 active diabetic users of insulin analogues related to sex, age, duration of diabetes diagnosis and insulin dose. [file 13098_2015_61_MOESM1_ESM.docx]

Table 1. Distribution of 215 active diabetic users of insulin analogues related to sex, age, duration of diabetes diagnosis and insulin dose

| Variables | Type 1 diabetes  n (%) | Type 2 diabetes (%) | Total  n (%) |
| --- | --- | --- | --- |
| Sex  Male  Female  Total | 36 (46.2)  42 (53.8)  78 (100) | 42 (30.7)  95 (69.3)*  137 (63.7) | 78 (36.3)  137 (63.7)  215 (100) |
| Age (years)  18 - 34  35 - 64  ≥ 65 | 43 (55.1)  35 (44.9)  0 | 1 (0.7)  71 (51.8)^†^  65 (47.5) | 44 (20.5)  106 (49.3)  65 (30.2) |
| Duration of diabetes diagnosis (years)  ≤ 10  11 - 20  ≥ 21 | 30 (38.5)  33 (42.3)  15 (19.2) | 29 (21.2)  60 (43.8)^‡^  48 (35) | 59 (27.5)  93 (43.2)  63 (29.3) |
| Dose of glargine (units) #  ≤ 30  31 - 60  ≥ 61 | 30 (39.4)  43 (56.5)  3 (3.9) | 34 (24.8)  70 (51.8) ^\|\|^  31 (22.9) | 64 (20.3)  113 (53.5)  34 (16.1) |
| Dose of short acting (units)^**^  ≤ 30  31 - 60  ≥ 61 | 52 (88.1)  6 (10.1)  1 (1.6) | 67 (69.7)  27 (28.1)^¶^  2 (2.08) | 119 (76)  33 (2.2)  3 (1.9) |

Regarding Type 1 diabetes (DM1): ^*^p = 0.02; ^†^p = 0.00001; ^ǂ^ p = 0.01; ^||^p = 0.02; ^¶^p = 0.007.

# Total = 211 (98.1%). DM1 = 74 patients (2, only short acting analogue). DM2 = 135 patients (2, short acting analogue only).

^**^ Total = 155 (72%). DM1 = 59 patients (17, dose not registered and 2 only glargine). DM2 = 96 patients (12, dose not registered; 29 glargine only).
