# Supplementary material for: Results of glycated hemoglobin during treatment with insulin analogues dispensed in the public health system of Federal District in Brazil
Source: Diabetol Metab Syndr. 2015 Aug 18;7:66. doi: 10.1186/s13098-015-0061-0 (PMC4539715; doi:10.1186/s13098-015-0061-0)
Supplement: Additional file 2: — Table S2. Mean, standard deviation (SD) of glycated hemogoblin (HbA1c) related to age, sex, type of insulin and targets to continuity of treatment for type 1 and type 2 diabetes. [file 13098_2015_61_MOESM2_ESM.docx]

**Table 2.** Mean, standard deviation (SD) of glycated hemogoblin (HbA1c) related to age, sex, type of insulin and targets to continuity of treatment for type 1 and type 2 diabetes

| Variables | Type 1 diabetes | Type 2 diabetes |
| --- | --- | --- |
| Age (years)  18 - 65  > 65 | 9.1 ± 1.7  0 | 9.0 ± 1.7  8.5 ± 1.1 |
| Sex  Male  Female | 8.7 ± 1.5  9.4 ± 1.7 | 8.3 ± 1.5  9.4 ± 1.4 |
| Insulins  Combination  Glargine  Short acting | 9.0 ± 1.5  10.3 ± 0.5  11.3 ± 4.5 | 8.9 ± 1.4  8.2 ± 1.5  6.3 ± 1.5 |
| Targets of continuity  *In the target**  Total  Female  Male  *Out of target*^†^  Total  Female  Male  *0.5% minimum reduction*  Total  Female  Male | 6.5 ± 0.4  6.4 ± 0.1  6.5 ± 0.5  9.4 ± 1.3  9.4 ± 1.4  9.5 ± 1.3  9.8 ± 1.6  10.0 ± 1.9  9.5 ± 1.3 | 6.7 ± 0.7  6.9 ± 0.6  6.4 ± 0.7  9.9 ± 1.3  9.9 ± 1.2  10.0 ± 1.4  9.1 ± 0.8  9.3 ± 0.9  8.8 ± 0.6 |

*In the target**: HbA1c < 7% (18-65 years) or HbA1c < 8% (> 65 years)

*Out of target*^†^: HbA1c ≥ 7% (18-65 years) or HbA1c ≥ 8% (> 65 years)
