# Supplementary material for: Results of glycated hemoglobin during treatment with insulin analogues dispensed in the public health system of Federal District in Brazil
Source: Diabetol Metab Syndr. 2015 Aug 18;7:66. doi: 10.1186/s13098-015-0061-0 (PMC4539715; doi:10.1186/s13098-015-0061-0)
Supplement: Additional file 3: — Table S3. Frequency of type 1 and type 2 diabetes among female and male according to criteria of continuity of insulin analogue treatment, based in HbA1c range of values. [file 13098_2015_61_MOESM3_ESM.docx]

**Table 3.** Frequency of type 1 and type 2 diabetes among female and male according to criteria of continuity of insulin analogue treatment, based in HbA1c range of values

| Criteria of continuity | Type 1 diabetes  n (%) | Type 2 diabetes  n (%) | Total  n (%) |
| --- | --- | --- | --- |
| *In the target**  Total  Female  18 - 65 years  > 65 years  Male  18 - 65 years  > 65 years | 13 (16.7)  4 (5.1)  0  9 (11.5)  0 | 43 (31.4)  8 (5.8)  19 (13.9)  9 (6.6)  7 (5.1) | 56 (26.0)^‡§^  12 (5.6)  19 (8.8)  18 (8.4)  7 (3.3) |
| *Out of target*^†^  Total  Female  18 - 65 years  > 65 years  Male  18 - 65 years  > 65 years | 41 (52.5)  23 (29.5)  0  0  18 (23.0) | 62 (45.3)  27 (19.7)  20 (14.6)  3 (2.2)  15 (10.9) | 103 (48.0)^\|\|^  50 (23.2)  20 (9.3)  3 (1.4)  33 (15.3) |
| *0.5% minimum reduction*  Total  Female  18 - 65 years  > 65 years  Male  18 - 65 years  > 65 years | 24 (30.1)  15 (19.2)  0  9 (11.5)  0 | 32 (23.4)  12 (8.7)  8 (5.8)  6 (4.4)  6 (4.4) | 56 (26.0)  27 (12.6)  8 (3.7)  15 (7.0)  6 (2,8) |

n = 78 (DM1). n = 137 (DM2). n = 215 (total).

*In the target**: HbA1c < 7% (18-65 years) or HbA1c < 8% (> 65 years)

*Out of target*^†^: HbA1c ≥ 7% (18-65 years) or HbA1c ≥ 8% (> 65 years)

^‡^p = 0.001 *vs* total *Out of target* and total *0.5% minimum reduction of HbA1c*

^§^p = 0,001 *vs* total *Out of target*

^||^p = 0,001 *vs* *0.5% minimum reduction of HbA1c*
