# Supplementary material for: Results of glycated hemoglobin during treatment with insulin analogues dispensed in the public health system of Federal District in Brazil
Source: Diabetol Metab Syndr. 2015 Aug 18;7:66. doi: 10.1186/s13098-015-0061-0 (PMC4539715; doi:10.1186/s13098-015-0061-0)
Supplement: Additional file 4: — Table S4. Multivariate analysis without distinction of diabetes type, HbA1c with sex, age, insulin combination, total dose of insulin, duration of treatment, and quantity of exams in 186 diabetic patients. [file 13098_2015_61_MOESM4_ESM.docx]

**Table 4**. Multivariate analysis without distinction of diabetes type, HbA1c with sex, age, insulin combination, total dose of insulin, duration of treatment, and quantity of exams in 186 diabetic patients *

| Variables | Coeficient | p |
| --- | --- | --- |
| Sex | 0.63 | 0.03 |
| Age | -0.02 | 0.01 |
| Insulin combination | -0.04 | 0.92 |
| Total dose of insulin | 0.01 | 0.002 |
| Duration of treatment | -0.0008 | 0.92 |
| Quantity of exams | -0.26 | 0.01 |
| Constant | 9.68 | 0 |

* twenty nine patients without using short acting insulin analogue were excluded from multivariate study
